# Influence of presence and patterns of late gadolinium enhancement on left ventricular remodeling after heart transplantation

**DOI:** 10.1186/1532-429X-15-S1-P260

**Published:** 2013-01-30

**Authors:** Maria Fernanda Braggion Santos, Jan Simpfendörfer, Evangelos Giannitsis, Hugo A Katus, Henning Steen

**Affiliations:** 1 Universitatsklinikum Heidelberg, Heidelberg, Germany; 2 School of Medicine of Ribeirao Preto University of Sao Paulo, Sao Paulo, Brazil

## Background

The presence of late gadolinium enhancement (LGE) is related to cardiac remodeling and poorer prognosis in ischemic and non-ischemic cardiomyopathies. Non-invasive LGE- cardiovascular magnetic resonance (LGE-CMR) is able to detect infarct-typical and -atypical patterns of LGE after heart transplantation (HTX), mainly due to post HTX coronary artery vasculopathy (CAV) and myocarditis. In this study, we sought to describe the influence of the presence and patterns of both LGE forms on left ventricular (LV) remodeling after HTX.

## Methods

153 HTX patients were divided into 3 groups depending on the presence and pattern of LGE (group I: atypical pattern, group II: typical pattern and group III: no LGE). 146 patients received coronary angiography within 4 weeks after CMR for assessment of CAV. Vector-ECG gated 32-channel parallel imaging cine SSFP sequences for standard imaging plane and LV volumetry acquisition were acquired on a 1.5T Whole Body MRI scanner (Achieva 1.5T, Philips Medical Systems). LGE-CMR (Gadolinium:0.2 mmol/kg) imaging was performed and analyzed blindly by two experienced observers. Patients were divided according to the presence and patterns of LGE. CAV was divided into three groups:1=mild;2=moderate;3=severe post transplant coronary artery disease. Data were expressed as mean and standard deviation or percentage. Groups were compared using Chi Square for categorical data and ANOVA or Kruskal-Wallis tests for continuous variables. P-values ≤ 0.05 were considered statistically significant.

## Results

**Figure 1:**
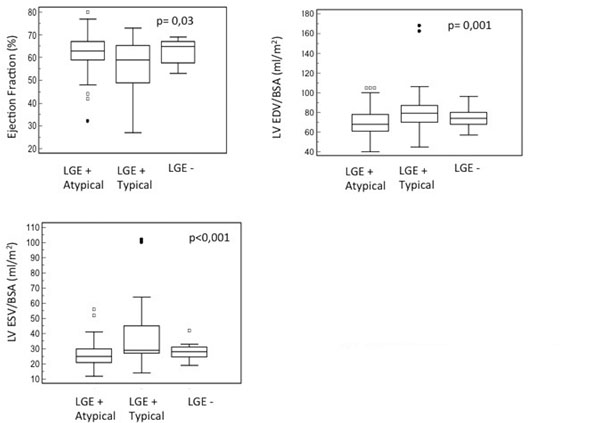
**Cardiovascular Magnetic Resonance Parameters in 3 Groups of HTX Patients according to the Presence and Pattern of LGE**. Box plots of Ejection Fraction, LV EDV/BSA and LV ESV/BSA in LGE + Atypical Pattern, LGE + Typical Pattern and LGE - Patients. LV= left ventricle; EDV= end-diastolic volume; ESV= end-systolic volume; BSA= body surface area; LGE+= presence of LGE; LGE- = absence of LGE.

Overall, 135 patients (88%) presented patterns of LGE, whereas infarct-atypical patterns were more prevalent (66%). Group II showed significantly lower ejection fractions (56±12%) when compared to group I (63 ± 8%) and III (63±5%;p=0,03) but significantly increased end-diastolic (83±26ml/m2) volumes/BSA (groupI=69±14ml/m2;III=75±10ml/m2;p=0,001) and end-systolic volumes/BSA (38±21ml/m2 vs. group I=26±8ml/m2 and III=28±5ml/m2; p<0,001, Figure 1). There was no significant difference related to LV morphology and function between groups II and III. Also, group II revealed less severe CAV presented with a smaller number of patients with mild coronary lesions (CAV1, p=0,02) and a larger number with severe lesions (CAV3, p<0,01).

## Conclusions

The presence of infarct-typical LGE-CMR is related to negative post HTX remodeling revealing lower ejection fractions and increased volumes in patients after HTX. Future studies are warranted to evaluate the long-term effect of infarct-typical and atypical patterns on future post HTX outcomes.

## Funding

none

